# Minimal Residual Disease-Based Risk Stratification in Chinese Childhood Acute Lymphoblastic Leukemia by Flow Cytometry and Plasma DNA Quantitative Polymerase Chain Reaction

**DOI:** 10.1371/journal.pone.0069467

**Published:** 2013-07-25

**Authors:** Suk Hang Cheng, Kin Mang Lau, Chi Kong Li, Natalie P. H. Chan, Rosalina K. L. Ip, Chi Keung Cheng, Vincent Lee, Matthew M. K. Shing, Alex W. K. Leung, Shau Yin Ha, Daniel K. L. Cheuk, Anselm C. W. Lee, Chak Ho Li, Chung Wing Luk, Siu Cheung Ling, Ondrej Hrusak, Ester Mejstrikova, Yonna Leung, Margaret H. L. Ng

**Affiliations:** 1 Department of Anatomical and Cellular Pathology, Prince of Wales Hospital, The Chinese University of Hong Kong, Hong Kong, China; 2 Department of Pediatrics, Prince of Wales Hospital, The Chinese University of Hong Kong, Hong Kong, China; 3 Department of Pediatrics, Queen Mary Hospital, Hong Kong, China; 4 Department of Pediatrics, Tuen Mun Hospital, Hong Kong, China; 5 Department of Pediatrics, Queen Elizabeth Hospital, Hong Kong, China; 6 Department of Pediatrics, Princess Margaret Hospital, Hong Kong, China; 7 Department of Pediatric Hematology and Oncology, 2nd Faculty of Medicine, Charles University, Prague, Czech Republic; 8 State Key Laboratory in Oncology in South China, The Chinese University of Hong Kong, Hong Kong, China; Westmead Millennium Institute, University of Sydney, Australia

## Abstract

Minimal residual disease, or MRD, is an important prognostic indicator in childhood acute lymphoblastic leukemia. In ALL-IC-BFM 2002 study, we employed a standardized method of flow cytometry MRD monitoring for multiple centers internationally using uniformed gating, and determined the relevant MRD-based risk stratification strategies in our local patient cohort. We also evaluated a novel method of PCR MRD quantitation using peripheral blood plasma. For the bone marrow flow MRD study, patients could be stratified into 3 risk groups according to MRD level using a single time-point at day-15 (Model I) (I-A: <0.1%, I-B: 0.1–10%, I-C: >10%), or using two time-points at day-15 and day-33 (Model II) (II-A: day-15<10% and day-33<0.01%, II-B: day-15≥10% or day-33≥0.01% but not both, II-C: day-15≥10% and day-33≥0.01%), which showed significantly superior prediction of relapse (p = .00047 and <0.0001 respectively). Importantly, patients with good outcome (frequency: 56.0%, event-free survival: 90.1%) could be more accurately predicted by Model II. In peripheral blood plasma PCR MRD investigation, patients with day-15-MRD≥10^−4^ were at a significantly higher risk of relapse (p = 0.0117). By multivariate analysis, MRD results from both methods could independently predict patients’ prognosis, with 20–35-fold increase in risk of relapse for flow MRD I-C and II-C respectively, and 5.8-fold for patients having plasma MRD of ≥10^−4^. We confirmed that MRD detection by flow cytometry is useful for prognostic evaluation in our Chinese cohort of childhood ALL after treatment. Moreover, peripheral blood plasma DNA MRD can be an alternative where bone marrow specimen is unavailable and as a less invasive method, which allows close monitoring.

## Introduction

Despite recent advances in the treatment of childhood acute lymphoblastic leukemia (ALL), the disease remains a major cause of cancer-related mortality in children. There are still around 20% of patients who develop relapse, and those who survive suffer from significant treatment related toxicities [1], [2], which reflects both under- or over-treatment of the disease as a result of the lack of accurate assessment of the leukemic status. A better assessment and particularly of patient’s risk of relapse at an early stage after therapy, is thus needed to improve patient outcome. Conventionally, the initial peripheral blood (PB) and bone marrow (BM) blast count, and the biological features of leukemic cells such as karyotypic abnormalities and immunophenotypes, have been adopted for risk stratification of ALL patients at diagnosis [3]. In the recent decades, detection of minimal residual disease (MRD) has become an important assessment and the level of MRD emerged as one of the most powerful indicators of treatment response in vivo, as it corresponds to the resistance of leukemic cells to chemotherapy and also the dynamic interactions of complicated therapy[4]–[6].

A variety of methods have been developed to detect MRD. The most commonly used are PCR analysis of immunoglobulin/T-cell receptor (Ig/TCR) gene rearrangements and flow cytometry (FCM)[7]–[11]. The FCM method has the advantage over PCR of providing a faster result and is more cost-effective and widely applicable to most patients. It can also be applied in multi-center setting using standardized reagents and gating strategies [12], [13]. Here we report our local findings as part of the I-BFM Mini-mini project using FCM for quantitation of MRD [12], and determined the clinically relevant MRD cutoffs for risk stratifying Chinese patients treated under ALL IC-BFM 2002 protocol.

Development of novel methods for MRD detection, especially by means of non-invasive sampling technique, provides an alternative approach to determine early treatment response in children. A number of studies have been done on the use of detecting free circulating nucleic acids in blood plasma as a tool for screening and disease monitoring of cancer patients[14]–[17]. BM is still the major source of material needed for MRD studies in childhood ALL, but the aspiration procedure is invasive for children and frequent disease monitoring is not possible. In this study, we evaluated the feasibility of MRD measurement on PB plasma DNA for quantitative PCR, and determined the prognostic significance of using this novel method for assessment of childhood ALL patients.

## Materials and Methods

### Ethics Statement

Informed written consent for the study was obtained from all patients’ parents/guardians, and the study was approved by the Joint CUHK-NTEC Clinical Research Ethics Committee.

### Patients

From January 2003 to April 2008, a total of 175 children (aged 1 to 18 years) diagnosed with ALL from 5 major hospitals in Hong Kong were recruited in the ALL IC-BFM 2002 study [12]. Patients recruited from January 2003 to December 2004 (n = 67) were assessed using 3-color panel and patients recruited from January 2005 to December 2009 (n = 139) were assessed using 4-color panel for FCM MRD. In addition 31 patients were recruited into another study (ALL CCLG-2008) from May 2008 to December 2009, and MRD was assessed using the same 4-color FCM panel. Follow up data and survival status was obtained up to August 2011. The median follow-up for survivors was 70.8 (ALL IC-BFM 2002) and 26.0 months (ALL CCLG-2008). Clinical characteristics of the patients were summarized in Table 1. The BM and PB collection was performed at diagnosis, day 15 of induction, end of induction phase I (day 33), and pre-consolidation (week 12). FCM MRD prognostic analysis was performed on 113 patients at day 15 and 109 patients at day 33 with sufficient/available BM materials.

**Table 1:** Summary of clinical and biological features for patients in the study.

Treatment protocol	ALL-IC-BFM 2002	ALL CCLG-2008
No. of patients	175	31
	***Median (range)***	***Median (range)***
Follow-up months	70.8 (36.8–101.7)	26.0 (17.2–36.8)
Age (years)	5.6 (1.1–17.9)	6.3 (1.2–16.8)
WBC (/1000 uL)	10.2 (0.7–673.8)	13.0 (1.5–384.5)
	***Frequency (%)***	***Frequency (%)***
**Sex**		
*male*	105 (60.0)	17 (54.8)
*female*	70 (40.0)	14 (45.2)
**Immunophenotype**		
*common*	99 (56.6)	17 (54.8)
*early B*	1 (0.6)	0 (0)
*mature B*	1 (0.6)	0(0)
*pre-B*	58 (33.1)	8 (25.8)
*pro-B*	2 (1.1)	0(0)
*T/pre-T*	14 (8.0)	6 (19.4)
**ALL-IC-BFM 2002/CCLG-2008 risk group**		
*standard risk*	60 (34.3)	18 (58.1)
*intermediate risk*	91 (52.0)	8 (25.8)
*high risk*	24 (13.7)	5 (16.1)
**Fusion genes**		
*BCR/ABL*	5 (2.9)	0 (0)
*MLL/AF4*	0 (0)	0 (0)
*TEL/AML1*	30 (17.1)	7 (24.1)
**Day 8 prednisone response**		
*good*	160 (91.4)	26 (83.9)
*poor*	15 (8.6)	5 (16.1)
**Outcome**		
*Complete remission*	155 (88.6)	29 (93.5)
*Death in induction*	2 (1.1)	2 (6.5)
*Relapse*	27 (15.4)	0 (0)
*Bone marrow transplant*	20 (11.4)	0 (0)
*Death after induction*	18 (10.3)	0 (0)

### Treatment

The treatment scheme for ALL IC-BFM 2002 protocol was reported previously [12]. In brief, after 7-day steroid monotherapy and one dose of intrathecal methotrexate (MTX), the patients received 8-week induction and early intensification therapy. Consolidation therapy consisted of high dose MTX and followed by re-induction with combinations consisting dexamethasone, vincristine, cyclophosphamide and anthracyclines. Cranial irradiation was applied in high-risk and T-ALL patients only. Maintenance therapy consisting of 6-mercaptopurine and MTX then continued up to 24 months. The risk group was defined according to standard BFM criteria, and MRD assessment was not applied for risk stratification.

ALL CCLG-2008 was similar to the previous protocol, but standard risk (SR) received a shortened early intensification therapy and less intensive re-induction. Cranial irradiation was applied only on patients with CNS disease or of very high risk not planning for BM transplant. The study used NCI criteria together with prednisone response for risk group stratification, and MRD was employed to stratify patients.

### Diagnostic Studies

The diagnosis of ALL was based on standard morphologic, cytochemical, immunophenotypic and genetic studies. Flow cytometric immunophenotyping of BM aspirates at diagnosis was performed using a standard panel of antibodies (HLA-DR, CD10, CD19, CD20, CD22, CD2, CD3, CD5, CD7, TdT, CD13, CD14, CD33, MPO, CD41, CD16, CD34, CD79a, glycophorin A, CD15) (Immunotech, France). The presence of fusion genes including TEL/AML1, BCR/ABL and MLL/AF4 was examined by PCR and fluorescence in situ hybridization. Conventional karyotyping was done using standard methods [18], and the karyotypes were described according to the International System of Human Cytogenetic Nomenclature [19].

### FCM MRD Analysis

Standardized operating procedures according to the Mini-mini study were adopted for sample preparation, data acquisition, and data analysis [12]. Leukemia-associated immunophenotypes (LAIPs) were studied at diagnosis on erythrocyte-lysed BM samples using the following antibody combinations: 3-color - CD19/CD10/CD20, CD19/CD10/CD33, CD19/CD10/CD66c for B lineage ALL; CD3/CD5/CD7 for T lineage ALL; 4-color - CD58/CD10/CD19/CD34, CD20/CD10/CD19/CD34, and CD66c/CD10/CD19/CD45 for B lineage ALL; CD99/CD7/CD3/CD5 and CD7/sCD3/cyCD3/TdT for T lineage ALL (BD Biosciences, San Jose, CA) (Immunotech). The same antibody combinations were applied during follow up for MRD quantitation. The strategy for MRD detection was based on detection of cells expressing an aberrant immunophenotype including asynchronous antigen expression and antigen overexpression compared to normal BM. Data acquisition was performed with FC 500 cytometer (Beckman Coulter, Miami, FL) and FlowJo (Tree Star, Ashland, OR) was used for data analysis. At least 20,000 events were acquired and analyzed for identification of aberrant leukemic phenotypes at diagnosis, and at least 300,000 events were needed for MRD measurements. A detection limit of 0.01% (10/100,000 cells) could be achieved in most cases.

### DNA Preparation and RQ-PCR MRD Analysis

Mononuclear cells (MNCs) from diagnostic BM samples were obtained using Ficoll-Paque density centrifugation (GE Healthcare, Sweden). Plasma samples were collected from diagnostic and follow-up PB. They were separated from PB within 24 hours after collection by centrifugation (3000×g, 10 minutes for 2 times). Genomic DNA was isolated by QIAamp DNA Blood kit (Qiagen, Germany) and QIAamp DNA Micro kit (Qiagen) for MNCs and plasma samples respectively. For quantitation of total plasma DNA and quality control in our samples, we performed beta-globin quantitative PCR for all the plasma DNA samples using method by Lo et al [20], and all samples have beta-globin being detected with a median level of 332.51±60.12 ng/ml. The input volume of plasma (0.4–0.8 ml) and the elution volume (30 ul) of DNA were standardized for later calculations.

The identification of PCR targets and RQ-PCR analysis were described previously [9]. In brief, multiplex PCR was performed using primers for detection of IgH, Igκ, T-cell receptor γ, and T-cell receptor δ gene rearrangements. Clonal bands were excised and sequencing was performed with 3100×L Genetic Analyzer (Applied Biosystems, Foster City, CA). Patient-specific primers for RQ-PCR were then designed according to the sequence analysis using IMGT database (http://imgt.cines.fr, IMGT, European Bioinformatics Institute, France). RQ-PCR was performed using 7300 real-time PCR system (Applied Biosystems). Five microliters of plasma-extracted DNA was used in each of the 20 ul RQ-PCR reactions, and performed in triplicates. DNA copy number of PB plasma samples was computed according to standard curves by serial dilutions of diagnostic BM samples. The sensitivity of plasma DNA MRD was tested by spiking DNA at different concentrations into plasma, then extracted DNA from these plasma samples and performed RQ-PCR (Figure S1).

### Statistical Analysis

Comparisons of categorical and continuous variables were performed using Fisher’s exact test and Mann Whitney U test, respectively. Risk of relapse or event-free survival (EFS) was estimated with Kaplan-Meier analysis and log-rank test. Relapse risk assessment was from the day of diagnosis to the occurrence of relapse. EFS was measured from the day of diagnosis until failure to achieve complete remission, transplant, relapse or death. Cox regression analysis was performed to test for the significance of MRD risk stratifications, controlling for potential prognostic factors including age at diagnosis, sex, B or T lineage, white blood cell count (WBC), hyperdiploidy, BCR-ABL translocation, TEL-AML1 translocation, ALL-IC-BFM 2002 risk group, treatment by bone marrow transplantation and day 8 prednisone response. A p-value of <0.05 was considered statistically significant. All calculations were performed with SPSS v17 (SPSS Inc., Chicago, IL).

## Results

### Leukemic-associated Immunophenotype (LAIP) Identification for FCM MRD Monitoring

#### Three-color FCM MRD

We recruited 67 patients for 3-color FCM MRD assessment, and 47 of them have available sufficient marrow specimens for diagnostic/follow-up study. In 35 of the 43 of B-lineage ALL patients at diagnosis, a population of leukemic cells could be identified by at least one LAIP, constituting a coverage rate of 81.4% (35/43). Among these 35 patients of B-ALL with positive LAIP, only 22.9% (8/35) had 2 or more while the majority 77.1% (27/35) had only 1 LAIP identified for follow-up. The most applicable antibody combination was CD19/CD10/CD20 (62.9%, 22/35), followed by CD19/CD10/CD66c (54.2%, 19/35), and CD19/CD10/CD33 (11.4%, 4/35). Single tube was used in T-ALL (CD3/CD5/CD7) with a coverage rate of 100% (4/4).

#### Four-color FCM MRD

With an aim to improve the coverage rate (≥1 LAIP) and the accuracy (with ≥2 LAIPs for each identifiable case) of MRD assessment, we switched to 4-color panel of FCM MRD assessment on 139 patients (122 of them had available BM material for MRD assessment). Although the coverage rate for B-ALL (82.4%, 89/108) was very similar to that obtained from 3-color panel, notably, there was a marked improvement of MRD quantitation in that 84.3% (74/89) of these patients had 2 or more LAIPs identified for follow up. For B-ALL, both CD20/CD10/CD19/CD34 and CD66c/CD10/CD19/CD45 were frequently expressed (74.2%, 66/89), and CD58/CD10/CD19/CD34 was slightly less common (65.2%, 58/89). Again, in T-ALL, the coverage rate was 100% and all patients (14/14) had 2 LAIPs for follow-up (CD99/CD7/CD3/CD5 and CD7/sCD3/cyCD3/TdT).

### Correlation of FCM MRD and Clinical/Biological Factors

We compared the clinical and biological profiles of patients assessed by 3-color and 4-color under ALL IC-BFM 2002 study and found no statistically significant differences in age and sex distribution, immunophenotypes, blood white cell counts, presence of fusion genes, karyotypes, risk group, prednisone response and treatment outcome (data not shown). Moreover, the proportions of patients with MRD <0.01% (3-color vs 4-color, Day33∶63.9% vs 44.9%, Week 12∶96.4% vs 81.7%) and their median MRD level at all time points were also comparable (Day 15∶0.13% Vs 0.19%, Day 33: <0.01% Vs <0.01%, Week 12: <0.01% Vs <0.01%). Thus the MRD data from these groups were pooled for the prognostic analysis. However In the ALL CCLG-2008 study assessed by 4-color panel, the median MRD level was higher (Day 15∶0.64%, Day 33∶0.56%). As this group of patients also had a short follow up (26.0 months) we did not include them for the prognostic analyses.

We then investigated the relationships of MRD levels at day 15 and day 33 among different clinical/biological factors (week 12 was not compared as most patients had MRD level below detection limit of 0.01%). In univariate analysis, we observed a significant association of higher MRD level in T-lineage ALL patients (day 15: p<0.001, day 33: p<0.001), ALL IC-BFM 2002 high risk group (day15: p<0.001, day33: p = 0.001), patients with poor day 8 prednisone response (day15: p<0.001, day 33: p<0.001), non-hyperdiploid ALL (day 15: p = 0.031, day 33: p<0.001), age >6 years (day15: p<0.001, not significant for day 33), and WBC >20000/ul (day 15: p = 0.049, not significant for day 33) (Figure S2).

### Determination of Clinically Relevant FCM MRD Cutoffs at Day 15 and Day 33

We divided the patients into high MRD (≥cutoff) or low MRD (<cutoff) groups according to different cutoffs and looked into their relationships with the relapse rate. As shown in Table 2, MRD>0.1% at day 15 showed some predictive value for relapse (p = 0.042) which improved at higher MRD cutoffs MRD>1% and MRD>10% (p = 0.001 and p<0.001 respectively). At day 33, patients with MRD level higher than 0.01% also had a significantly higher relapse rate (p = 0.004), but the specificity (64.0%) in predicting patients in remission by Day 33 MRD <0.01% was lower than that by using the cutoff of 1% or 10% at day 15 (74.4% and 92.2% respectively) (Table 2). An analysis of outcome according to levels of MRD on day 33 only was also shown (Figure S3).

**Table 2:** Prognostic significance of FCM MRD at different cutoffs.

Time point: MRD cut-off		Relapse				Sensitivity	Specificity	Positive predictivevalue	Negative predictivevalue	p-value (Fisher’s exact)
		no		yes						
Day 15∶10%	≥10%	7	(7.8%)	9	(47.4%)	47.4%	92.2%	56.3%	89.2%	<0.001
	<10%	83	(92.2%)	10	(52.6%)					
Day 15∶1%	≥1%	23	(25.6%)	13	(68.4%)	68.4%	74.4%	36.1%	91.8%	0.001
	<1%	67	(74.4%)	6	(31.6%)					
Day 15∶0.1%	≥0.1%	47	(52.2%)	15	(78.9%)	78.9%	47.8%	24.2%	91.5%	0.042
	<0.1%	43	(47.8%)	4	(21.1%)					
Day 15∶0.01%	≥0.01%	71	(78.9%)	16	(84.2%)	84.2%	21.1%	18.4%	86.4%	NS
	<0.01%	19	(21.1%)	3	(15.8%)					
Day 33∶1%	≥1%	5	(5.6%)	2	(10.5%)	10.5%	94.4%	28.6%	83.2%	NS
	<1%	84	(94.4%)	17	(89.5%)					
Day 33∶0.1%	≥0.1%	19	(21.3%)	8	(42.1%)	42.1%	78.7%	29.6%	86.4%	NS
	<0.1%	70	(78.7%)	11	(57.9%)					
Day 33∶0.01%	≥0.01%	32	(36.0%)	14	(73.7%)	73.7%	64.0%	30.4%	91.9%	0.004
	<0.01%	57	(64.0%)	5	(26.3%)					

### Superior Risk Prediction by FCM MRD Stratification Based on Both Day 15 and Day 33 MRD Values (Model II)

**Figure 1:**
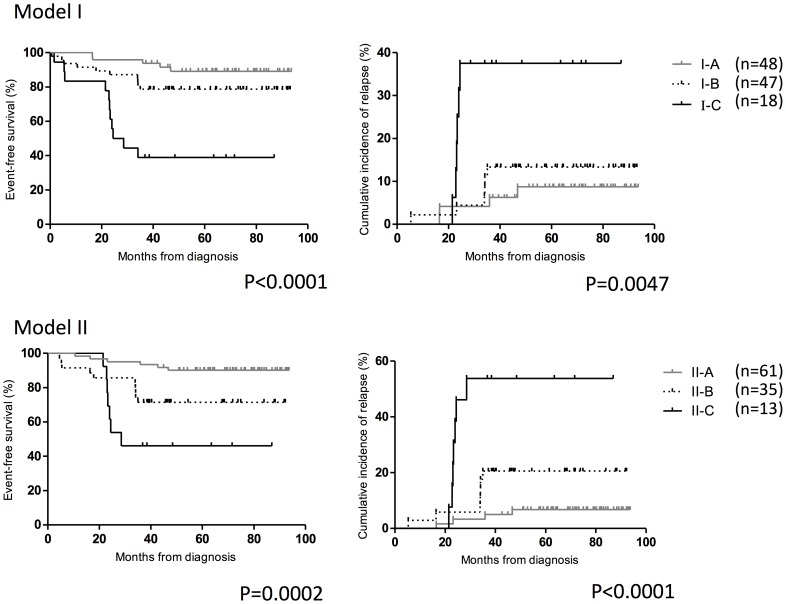
Prognostic significance of MRD as detected by FCM at day 15 and day 33. Event-free survival (left) and incidence of relapse (right) of patients stratified according to FCM MRD Model I (upper panel) and Model II (lower panel).

To apply the risk stratification scheme based on FCM MRD, we stratified the patients into three risk groups (I-A: <0.1%, I-B: 0.1–10%, I-C: >10%) according to MRD measured at day 15 (Model I). We found patients with high-level MRD (I-C) (n = 18, 15.9%) were significantly associated with low EFS and high incidence of relapse compared to those with intermediate MRD (I-B) (n = 47, 41.6%) and low MRD (I-A) (n = 48, 42.5%). The 5-year EFS and 5-year incidence of relapse for I-A vs I-B vs I-C were 89.1% vs 78.7% vs 38.9% (p<0.0001) and 8.7% vs 13.3% vs 37.5% (p = 0.0047) respectively (Figure 1, upper left and right).

As day 33 MRD>0.01% was highly predictive of relapse in the previous determination, to refine the risk stratification scheme based on FCM MRD, we designed another MRD risk group stratification (Model II) based on both day 15 and day 33 MRD values (II-A: day 15<10% and day 33<0.01%; II-B: day 15≥10% or day 33≥0.01% [but not both]; II-C: day 15≥10% and day 33≥0.01%). Likewise, patients with high MRD at both time points (II-C) (n = 13, 11.9%) had worse outcome compared to those who only had MRD above cutoff at one time point (II-B) (n = 35, 32.1%) or had both below cutoff (II-A) (n = 61, 56.0%), as evidenced by the 5-year EFS (II-A vs II-B vs II-C: 90.1% vs 71.4% vs 46.2%) (p = 0.0002) and 5-year incidence of relapse (II-A vs II-B vs II-C: 6.8% vs 20.6% vs 53.8%) (p<0.0001) (Figure 1, lower left and right). Comparing the figures of 5-year EFS and relapse rates from the two models, Model II showed superior prediction and better segregation of patients with different risks of relapse/survival.

### Significant Prognostic Impact of FCM MRD within B-lineage ALL Patients and ALL IC-BFM 2002-stratified Risk Groups

**Figure 2:**
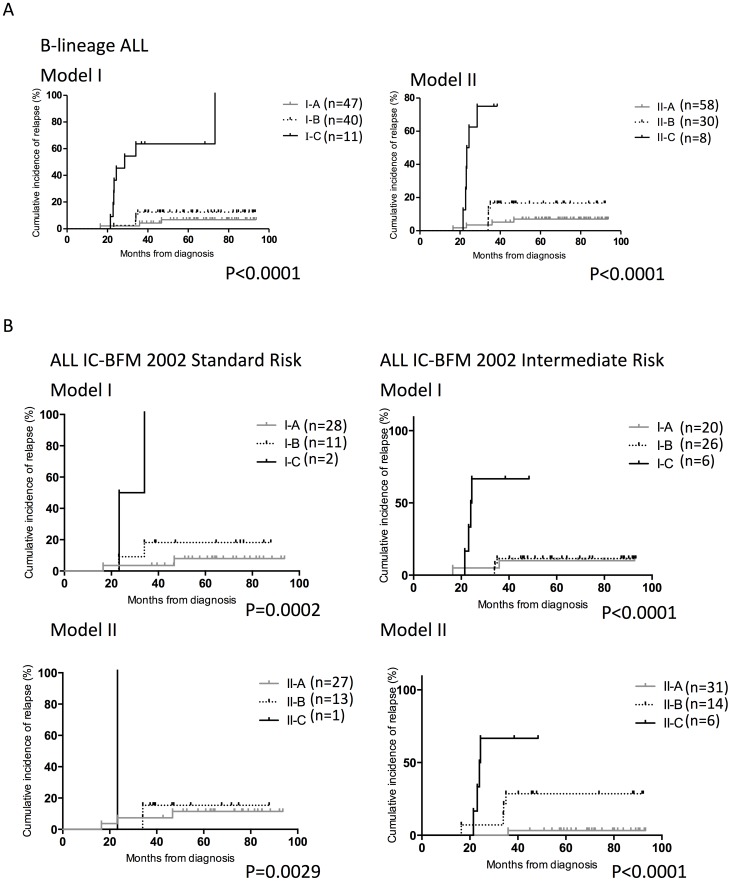
Prognostic significance of FCM MRD for patients with selected features. Incidence of relapse for patients with (A) B-lineage ALL (B) in ALL IC-BFM 2002 standard risk (left) or intermediate risk (right). Upper panels were stratified by Model I and lower panels were by Model II.

We further assessed the predictive value of FCM MRD by excluding T-lineage ALL patients which generally had poorer treatment outcomes. As illustrated in Figure 2A, both Model I and II maintained significant prognostic impact (p<0.0001 for both) by identifying patients with differential outcomes. In high risk (I-C and II-C) patients, the 5-year relapse rates were 63.6% and 75.0% respectively; whereas in low risk group (I-A and II-A), the rates were only 6.8% and 7.0%.

In ALL IC-BFM 2002 risk group classification, MRD values were not used as patient stratification criterion; thus it is of interest to assess the predictive value of MRD in these conventional risk groups. As shown in Figure 2B, patients originally defined as ALL IC-BFM 2002- Standard risk (SR) and intermediate risk (IR) could be further stratified by the FCM MRD models (high risk patients were not analyzed due to small sample size): For Model I, the 5-year incidence of relapse was 8.0% (I-A), 18.2% (I-B), and 100.0% (I-C) in BFM-SR (p = 0.0002); and 10.0% (I-A), 11.5% (I-B) and 66.7% (I-C) respectively in BFM-IR (p<0.0001). For Model II, the incidence was 11.4% (II-A), 15.4% (II-B), and 100.0% (II-C) in BFM-SR (p = 0.0029); and 3.2%, 28.6% and 66.7% in BFM-IR respectively (p<0.0001) (Figure 2B).

### PB Plasma DNA for MRD Monitoring: Feasibility and Prognostic Value

**Figure 3:**
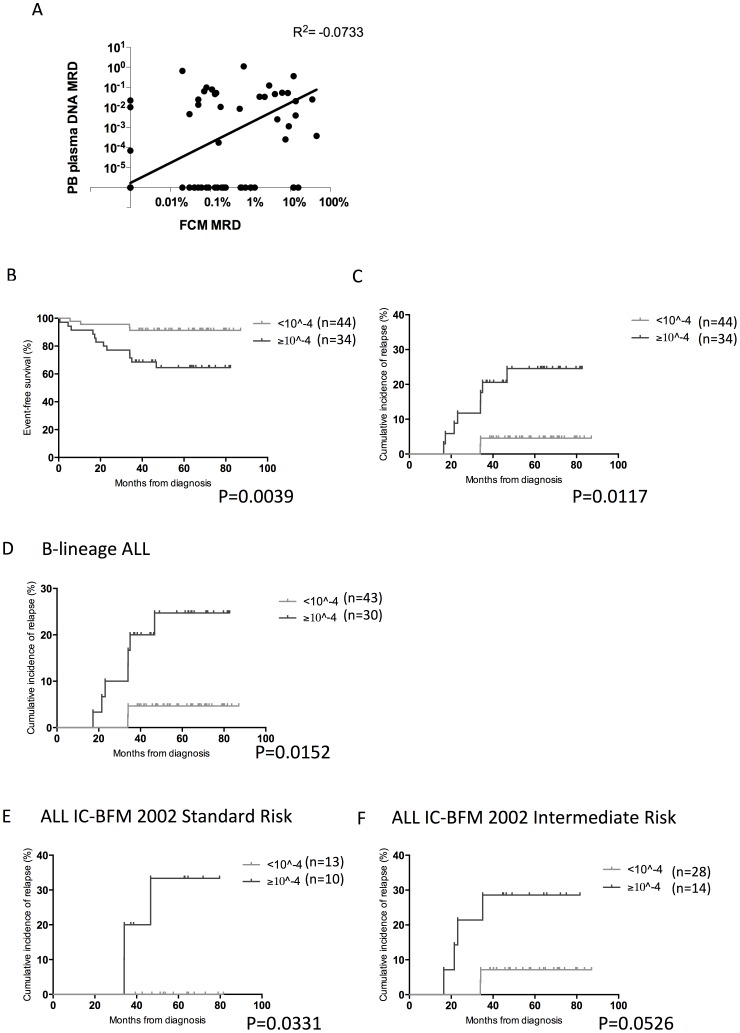
Prognostic significance of MRD as detected by PB plasma DNA PCR at day 15. (A) Correlation of PB plasma DNA MRD with FCM MRD at day 15. Spearman r test was used for statistical analysis. (B–C) Comparison of PB plasma DNA MRD level (cutoff: 10^−4^) at day 15 with (B) event-free survival and (C) occurrence of relapse. (D–F) Incidence of relapse for patients stratified according to PB plasma MRD cutoff at 10^−4^ with (D) B-lineage ALL, in ALL IC-BFM 2002 standard risk (E) or intermediate risk (F).

To assess the feasibility of MRD monitoring using DNA from PB plasma, we performed the quantitation of Ig/TCR gene rearrangements on plasma samples collected at day 15, day 33 and week 12 of treatment. Most samples collected at day 33 and week 12 had MRD below detection limit of 10^−5^ and were not analyzed further. We first compared the data of PB plasma DNA MRD with FCM MRD as shown in Figure 3A. Of 63 cases with available data for both MRD detection methods, only 35 of them (55.6%) were defined as MRD-positive/negative by both FCM (cutoff: 0.01%) and plasma DNA MRD (cutoff: 10^−5^). The 2 methods did not yield significant correlations in MRD levels (R^2^: −0.0733).

We then analyzed PB plasma DNA MRD data independently for its prognostic value. A significant correlation of day 15 MRD levels with the incidence of relapse was observed (n = 78, p = 0.04), in which the patients who eventually relapsed had a median MRD level of 4.26×10^−3^, compared to <10^−5^ in patients undergoing remission. The relationship of plasma DNA MRD data with clinical and biological profiles was also studied, but no significant association was observed (data not shown). To define an appropriate cutoff with prognostic significance, we again analyzed the data by dividing the patients into high or low MRD groups at MRD levels from 10^−2^ to 10^−5^ (Table 3). Cutoffs from 10^−3^ to 10^−5^ could significantly differentiate patients who developed relapse (sensitivity: 70.0–80.0%) and who were in remission (specificity: 58.8–67.6%), where the best cutoff was 10^−4^ (p = 0.018).

**Table 3:** Prognostic significance of PB plasma DNA MRD at different cutoffs.

Time point: MRD cut-off		Relapse				Sensitivity	Specificity	Positive predictive value	Negative predictive value	p-value (Fisher’s exact)
		no		yes						
Day 15∶10^−2^	≥10^−2^	17	(25.0%)	4	(40.0%)	40.0%	75.0%	19.0%	89.5%	NS
	<10^−2^	51	(75.0%)	6	(60.0%)					
Day 15∶10^−3^	≥10^−3^	22	(32.4%)	7	(70.0%)	70.0%	67.6%	24.1%	93.9%	0.034
	<10^−3^	46	(67.6%)	3	(30.0%)					
Day 15∶10^−4^	≥10^−4^	26	(38.2%)	8	(80.0%)	80.0%	61.8%	23.5%	95.5%	0.018
	<10^−4^	42	(61.8%)	2	(20.0%)					
Day 15∶10^−5^	≥10^−5^	28	(41.2%)	8	(80.0%)	80.0%	58.8%	22.2%	95.2%	0.038
	<10^−5^	40	(58.8%)	2	(20.0%)					

Using the cutoff at 10^−4^, patients with high MRD (n = 34) was associated with poorer 5-year EFS (64.5%) and higher incidence of relapse (24.6%) compared to patients with low MRD (n = 44) (EFS: 91.3%, relapse rate: 4.5%) (p = 0.0039 and p = 0.0117 respectively) (Figures 3B and C). It maintained a significant prognostic impact also in patients with B-lineage ALL, or within ALL IC-BFM-2002 SR and IR groups analyzed separately. In patients with B-lineage ALL the relapse rate was 24.7% for patients with MRD ≥10^−4^ (n = 73) and 4.7% for those with MRD <10^−4^ (p = 0.0152)(Figure 3D). For the BFM-SR group (n = 24) (Figure 3E), 33.3% with MRD ≥10^−4^ relapsed versus 0% in MRD <10^−4^ (p = 0.0331); and in BFM-IR (n = 42) (Figure 3F), the relapse rate was 28.6% vs 7.1% respectively (p = 0.0526).

### Multivariate Analyses of MRD Risk Stratification by FCM and PB Plasma PCR

The significance of MRD by FCM, and PCR using plasma DNA for predicting relapse was tested in Cox regression analyses (Table 4). Parameters included in the models with potential prognostic effect were age at diagnosis, sex, B or T lineage, WBC, hyperdiploidy, BCR-ABL translocation, TEL-AML1 translocation, ALL-IC-BFM 2002 risk group, day 8 prednisone response, and bone marrow transplantation (BMT). FCM MRD Models I and II, and PB plasma MRD at 10^−4^ on day 15 remained significant even after controlling for all the prognostic factors. Notably, patients having high-level FCM MRD had approximately 20 to 35-fold increase (p<0.001) in the risk of relapse, and those having plasma MRD of ≥10^−4^ had a 5.8 fold higher risk of relapse (p = 0.038).

**Table 4:** Multivariate Cox regression analysis for FCM and plasma DNA MRD.

Parameter	Hazard Ratio	95% CI	P-value
MRD on day 15 (Model I)			
*<0.1%*	1		
*0.1 to 10%*	2.584	0.607–10.995	<0.001
*>10%*	20.911	4.610–94.850	<0.001
MRD on two timepoints(Model II)			
*D15<10% and D33<0.01%*	1		
*D15≥10% or D33≥0.01%*	4.444	1.097–18.010	<0.001
*D15≥10% and D33≥0.01%*	35.182	6.821–181.471	<0.001
PB plasma PCR MRD on day 15			
*<10^−4^*	1		
*≥10^−4^*	5.800	1.101–30.544	0.038

Parameters included in the analysis but not significant:

Sex, B/T lineage, age at diagnosis, WBC count, BCR-ABL, TEL-AML1 translocation, ALL-IC-BFM 2002 risk group, day 8 prednisone response, BMT.

## Discussion

MRD detection has become an essential tool for assessment of in vivo treatment response in childhood ALL and FCM is one of the most commonly used technologies, which is easy to apply and with fast available results. The standardization of FCM MRD method which has taken place in several centers has demonstrated reproducibility with a good correlation to patient’s treatment outcome[12], [13], [21]–[23], thereby favoring its application in multicenter studies.

Our study was also derived from a multicenter international collaboration using standardized methodology of FCM MRD (Mini-mini study). We previously demonstrated the feasibility of uniform method for FCM MRD detection applied in multi-centers [12]. Here we presented our local data and extended our observations on its relationships to treatment outcome. We established two MRD risk stratification strategies, one using MRD data from single time point at day 15 (Model I) and the other using two time points at day 15 and day 33 (Model II). Patients could be stratified according to FCM MRD risk groups, with a significantly high incidence of relapse for group I-C and II-C (37.5% and 53.8%) but only 8.7% and 6.8% in I-A and II-A. Both models were still applicable if cases with known risk factors were removed (T-lineage ALL, ALL IC-BFM 2002 high risk). In particular, our FCM MRD risk stratifications were the most potent prognostic factors among all the conventional indicators and BM PCR MRD (unpublished in-house data).

Our findings were in line with those from other prognostic studies on FCM MRD. The Children’s Oncology Group showed that in patients positive (≥0.01%) for day-29 (end-of-induction) MRD was associated with poorer outcome (p<0.0001) compared to those with negative MRD, and day-29 MRD was the independent prognostic variable in multivariate analysis [24]. The group from St. Jude’s Children’s Research Hospital studied day-19 of induction therapy and showed that patients with ≥0.01% residual leukemic cells had significantly higher incidence of relapse/failure to achieve remission than those with undetectable leukemic cells (p<0.001) [10]. A recent AIEOP-BFM-ALL 2000 study, using similar treatment regimens and antibody panels with ours, stratified similar risk groups according to FCM MRD at day 15 as our Model I and found significant difference in incidences of relapse among the 3 risk groups (p<0.001). The risk groups were also found to be the most potent prognostic factor in multivariate analysis, with 2 to 5-fold increases in risk of relapse for patients classified into medium and high risk groups [21]. In addition to these similar findings, we demonstrated the prognostic impact of another FCM MRD risk stratification strategy using MRD results from two time points (Model II). This model provided a better discrimination of patients than FCM Model I stratification with significantly increasing risk of relapse in higher risk groups (II-A vs B vs C: 6.8% vs 20.6% vs 53.8%) (p<0.0001), which may better reflect the status of leukemic cell clearance as MRD at both time points were considered. Furthermore, Model II could better identify patients with good outcomes. The I-A and II-A have similarly good EFS (89.1% and 90.1%) but the percentage of patients being in good risk increased from 48/113 (42.5% by Model I) to 61/109 (56.0% by Model II), indicating that more patients could be accurately predicted to have good outcome and may benefit from reduced treatment intensity. In conclusion, both Model I and II have similar specificities in identifying good risk patients (IA or IIA). However, Model II has a higher sensitivity to identify these good risk patients (IIA) and these patients had been mis-classified as intermediate risk (IB) or to a lesser extent, as high risk (IC) in Model I. It would thus be advisable that group IB or IC patients (by Model I) should be tested beyond day 15 for more accurate risk classification by Model II.

With the stratification based on ALL IC-BFM 2002 (Figure 2B), the Model II could not further refine the risk groups in SR patients, as the incidence of relapse were similar in both I-A versus II-A and I-B versus II-B, and the percentage of patients distributed in the 3 risk groups in Model I and II were also similar in SR. However, the Model II had a significant effect on relapse prediction in IR group. Model II identified low risk patients with only a 3.2% relapse rate compared to a 10% rate in Model I. More importantly, the percentage of patients in IR predicted to have good outcome increased from 20/52 (38.5% by Model I) to 31/51 (60.8% by Model II). We could also identify a subset of IR patients with higher risk of relapse by Model II (II-B with 28.6% versus I-B with 11.5%), and this may justify further intensification of treatment in this subset of patients. Thus, the Model II prediction might benefit the BFM IR group but may not be beneficial to SR group.

It is interesting that we found the applicability of antibody combinations were different with other reported studies. Aberrant expression of CD66c was observed in 43% of European patients and 10–20% of Americans [10], [25], but it was more frequently expressed in our cohort (54.2% for 3-color and 74.2% for 4-color). CD58 was overexpressed in only 33.3% of patients in Patkar’s study [26], but we found more frequent aberrant expression at 65.7%. Similar frequency was found in CD10-CD20 combinations (71.4% by Patkar et al [26] and 62.9–74.2% by us). Thus, our observations suggested that the usefulness of antibody combinations may depend on patient’s ethnic group, and evaluation of antibodies for MRD detection should be done independently in each center. Besides, the coverage rate of FCM MRD for B-lineage ALL patients was around 80%, which was less desirable compared to the other studies with >90%(10–11). Therefore, we currently adopted an extended panel of antibodies to aim for a better applicability of FCM MRD to most patients, and our preliminary results showed an improvement of coverage to >95% (unpublished data).

In the second part of the study, we evaluated a non-invasive method of MRD detection using PB plasma for PCR. This is, to our knowledge, the first study to quantify Ig/TCR gene rearrangements in PB plasma DNA of childhood ALL patients by adopting the technique on BM to PB plasma DNA. A few studies have attempted to use plasma DNA for MRD detection, but they were limited to measure the concentration of plasma DNA or to detect the PCR products qualitatively [27], [28]. In fact, detection of circulating DNA in blood plasma has been studied in various cancers for early cancer detection and disease monitoring[14]–[17]. It was postulated that the origin of these circulating DNA was derived from tumor necrosis and apoptosis, and high concentrations of plasma DNA was found to be correlated to the larger tumor size and advanced diseases [29]. We found poor correlations of MRD levels by plasma DNA and FCM, which can be explained by the difference in source material being analyzed (PB cell-free DNA vs BM cells), and different leukemic targets being detected/quantified (with clonal IgH/TcR vs aberrant antigen expression). Nevertheless, both methods can independently predict relapse as demonstrated by multivariate analysis. The results of our study not only supported the idea and feasibility of using PB plasma DNA as a tool for treatment monitoring in leukemia patients, we also demonstrated the prognostic impact of PB MRD. Thus, MRD results from plasma DNA can be applied where BM specimens are not available, or for more frequent disease monitoring without the need for BM aspiration.

In this study, we have established MRD risk stratification strategies for Chinese childhood ALL patients under ALL IC-BFM 2002 protocol. The potential role of using PB plasma DNA as a non-invasive tool for MRD monitoring was also demonstrated. Further studies with larger patient cohort and earlier time points are warranted to clarify the clinical significance of using plasma DNA as an alternative tool for MRD monitoring.

## Supporting Information

Figure S1 The sensitivity of plasma DNA MRD by spiking experiment.
(TIFF)

Figure S2 The relationships of MRD levels at day 15 and day 33 among clinical/biological factors.
(TIFF)

Figure S3 An analysis of outcome according to levels of MRD on day 33 only.
(TIFF)
