# Supplementary figures and images for: Minimal Residual Disease-Based Risk Stratification in Chinese Childhood Acute Lymphoblastic Leukemia by Flow Cytometry and Plasma DNA Quantitative Polymerase Chain Reaction
Source: PLoS One. 2013 Jul 25;8(7):e69467. doi: 10.1371/journal.pone.0069467 (PMC3723913; doi:10.1371/journal.pone.0069467)

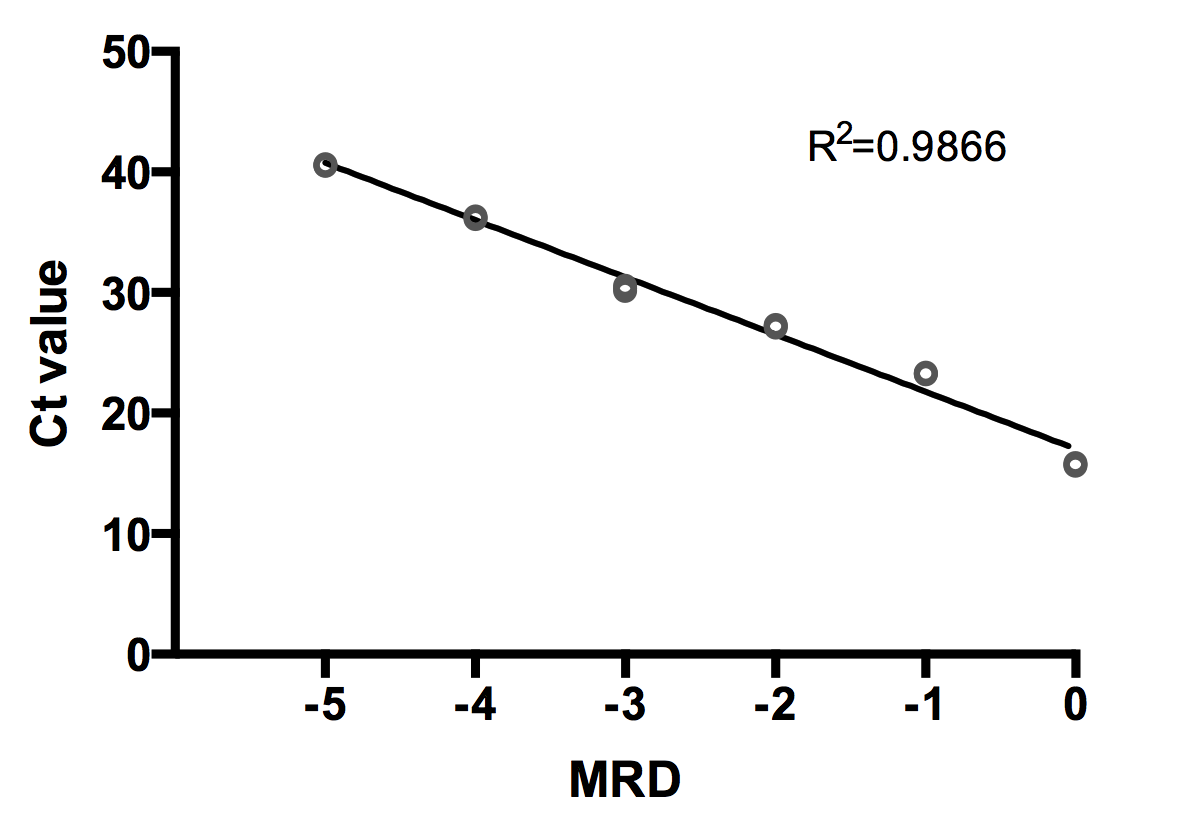

Supplement: Figure S1 — The sensitivity of plasma DNA MRD by spiking experiment. (TIFF) [file pone.0069467.s001.tiff]

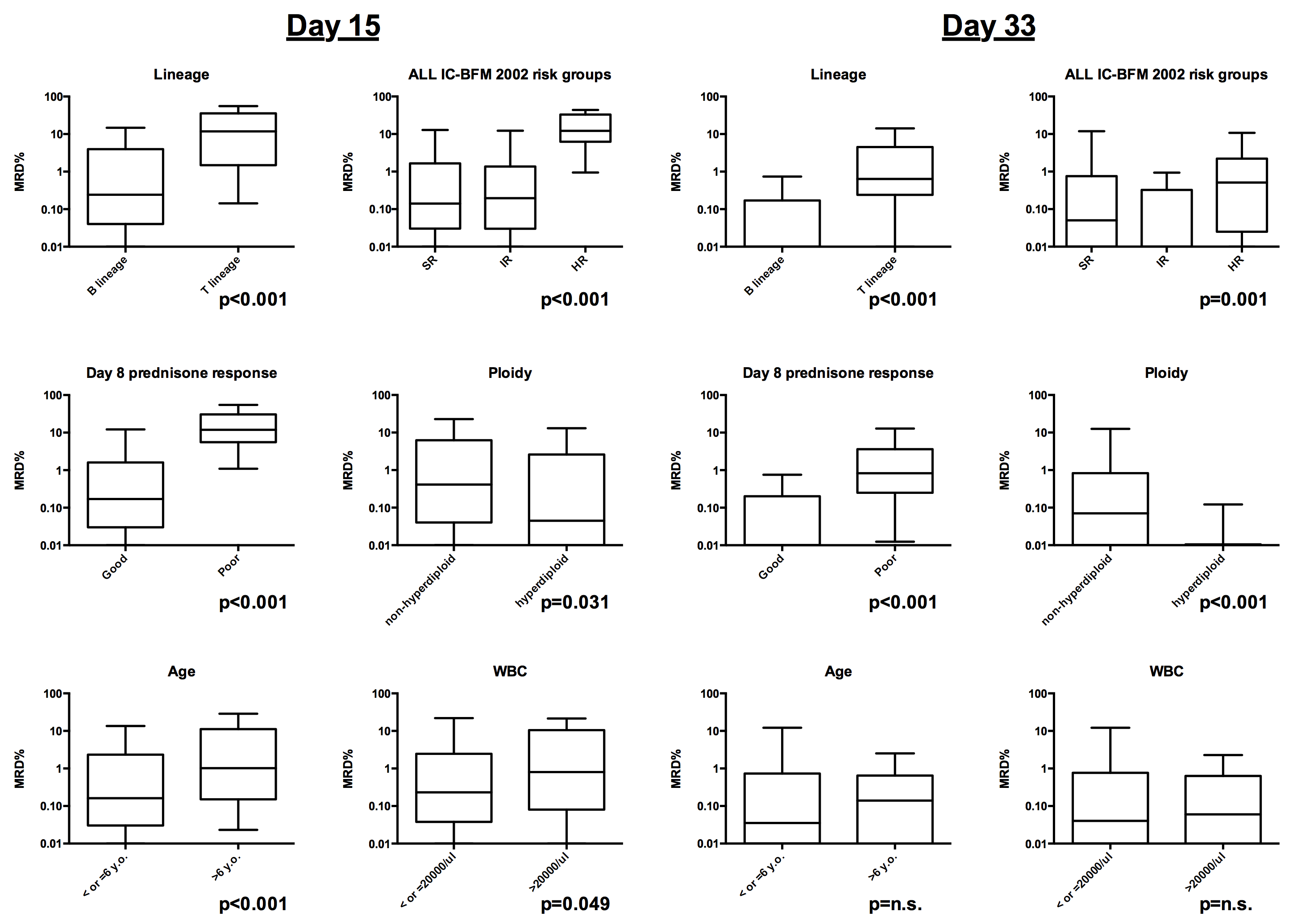

Supplement: Figure S2 — The relationships of MRD levels at day 15 and day 33 among clinical/biological factors. (TIFF) [file pone.0069467.s002.tiff]

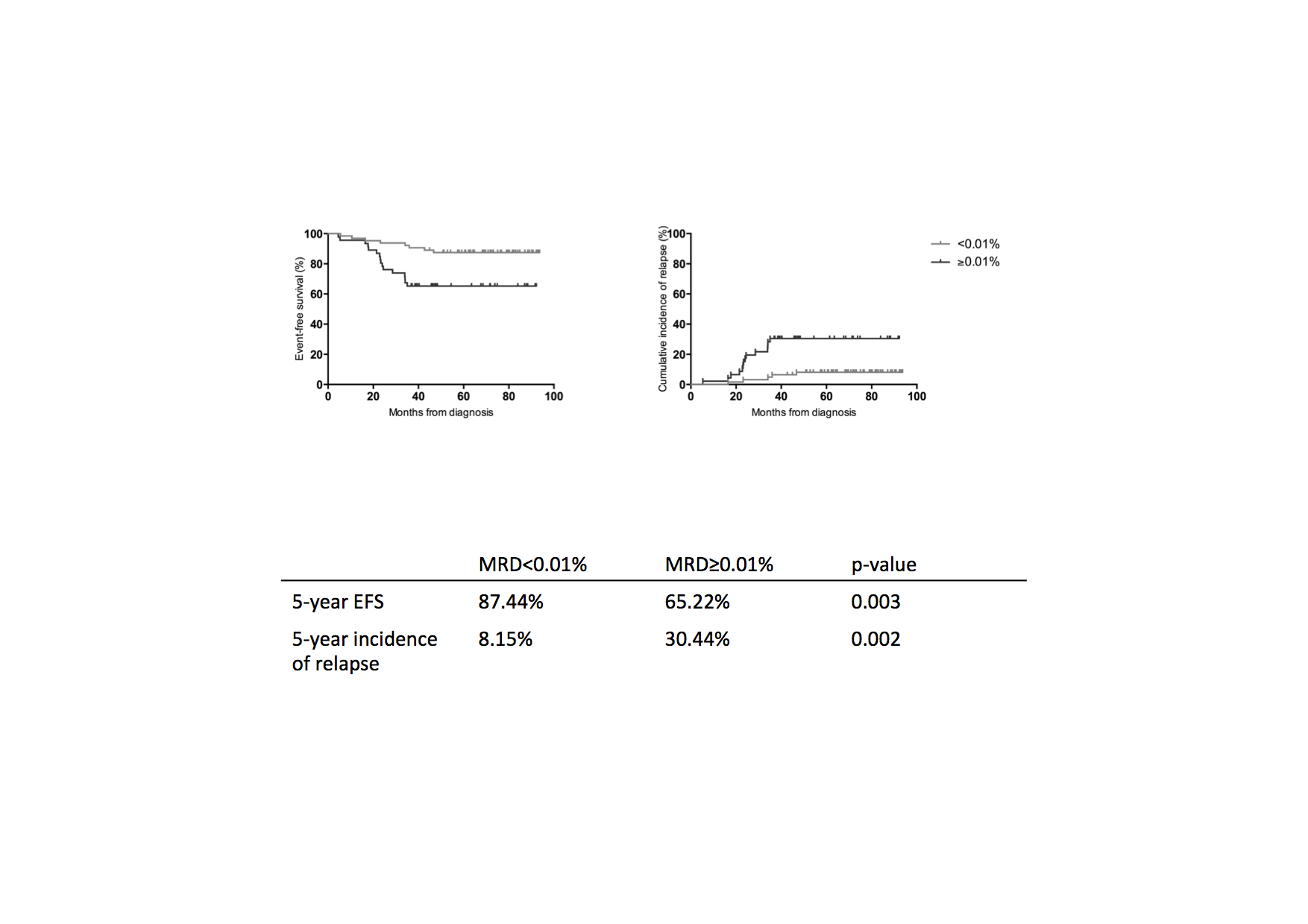

Supplement: Figure S3 — An analysis of outcome according to levels of MRD on day 33 only. (TIFF) [file pone.0069467.s003.tiff]
